# Subchronic Toxicity Studies of Cortex Dictamni Extracts in Mice and Its Potential Hepatotoxicity Mechanisms in Vitro

**DOI:** 10.3390/molecules23102486

**Published:** 2018-09-28

**Authors:** Qiongyin Fan, Baosheng Zhao, Chunguo Wang, Jingxuan Zhang, Jinying Wu, Ting Wang, Anlong Xu

**Affiliations:** Beijing Research Institute of Chinese Medicine, Beijing University of Chinese Medicine, Beijing 100029, China; bingofqy@163.com (Q.F.); zhaobs1973@163.com (B.Z.); wangcg1119@126.com (C.W.); zh_xyj@126.com (J.Z.); wujinying1212@sina.com (J.W.)

**Keywords:** Cortex Dictamni, hepatotoxicity, aqueous extract, ethanol extract, mechanisms, dictamnine, cell apoptosis

## Abstract

Cortex Dictamni is a commonly-used traditional Chinese herbal medicine for the treatment of skin inflammation, tinea, and eczema. Recently, some studies reported that Cortex Dictamni might induce liver injury, suggesting more attention to its safety. The current study was designed to investigate subchronic toxicity of Cortex Dictamni aqueous extract (CDAE) and ethanol extract (CDEE) in mice and the potential hepatotoxicity mechanisms in vitro. Firstly, CDAE or CDEE groups were administrated with varying dosages (2.3, 4.6, or 9.2 g/kg/day, p.o.) in mice for 28 days in subchronic toxicity studies. General clinical signs and biochemical parameters were examined, and morphological analyses were conducted. Secondly, we identified the different constituents of CDAE and CDEE using HPLC-MS/MS and chose major components for further study. In order to determine the toxic components, we investigated the cytotoxicity of extracts and chosen components using CCK-8 assay in HepG2 cells. Furthermore, we explored the possible hepatotoxicity mechanisms of Cortex Dictamni using a high content analysis (HCA). The results showed that no significant differences of general clinical signs were observed in mice. Aspartate alanine aminotransferase (ALT) and aminotransferase (AST) were significantly increased in the high-dose CDAE and CDEE groups compared to the control group. Meanwhile, the absolute and relative liver weights and liver/brain ratio were significantly elevated, and histological examination of liver demonstrated cellular enlargement or nuclear shrinkage. In UPLC analysis, we compared the chemical constituents between CDAE and CDEE, and chose dictamnine, obakunone, and fraxinellone for hepatotoxicity evaluation in the in vitro studies. In the CCK-8 assay, CDAE, CDEE, dictamnine, obakunone, and fraxinellone decreased the cell viability in a dose-dependent manner after treatment for 48 h. Furthermore, the cell number decreased, while the nuclear intensity, cell membrane permeability, and concentration of reactive oxygen species were shown to increase, meanwhile, mitochondrial membrane potential was also changed in HepG2 cells following 48 h of compounds treatment using HCA. Our studies suggested that CDAE and CDEE have potential hepatotoxicity, and that the alcohol extraction process could increase toxicity. Dictamnine, obakunone, and fraxinellone may be the possible toxic components in Cortex Dictamni with dictamnine as the most potentially hepatotoxic component, whose potential hepatotoxicity mechanism may be associated with cell apoptosis. Moreover, this study could provide valuable data for clinical drug safety research of Cortex Dictamni and a good example for safety study of other Chinese herbal medicines.

## 1. Introduction

Drug-induced liver injury (DILI) often results in the termination of drug development at the clinical stage or withdrawal of a drug from the market [1]. Compared with Western medicine, traditional Chinese medicine (TCM) has always been considered relatively safe and produces fewer adverse reactions in China [2]. However, in recent years, reports of adverse reactions have gradually increased with the expanded usage of TCM in various diseases. Particularly, some traditionally-considered nontoxic TCM herbs have been reported to cause serious adverse events (SAE) and acute deaths [3,4], suggesting a sparked concern for the safety of Chinese medicines. Thus, we here designed the research on potentially toxic TCM herbs [5].

Cortex Dictamni is a commonly-used TCM herbal medicine that originates from the dried root bark of *Dictamnus dasycarpus* Turcz, which has about six species and is widely spread in Europe and Asia. There are two species in China, *Dictamnus dasycarpus* Turcz and *Dictamnus angustifolius* G. Don, which are mainly distributed in Xinjiang, Henan, and northeast China [6]. According to the Pharmacopoeia of the People’s Republic of China and the principle of TCM, the function of Cortex Dictamni is to clear heat, dry dampness, dispel wind, and relieve toxicity, and it is used for the treatment of various illnesses, such as skin inflammation, tinea, rheumatoid arthritis, eczema, rubella, chronic hepatitis, coronary atherosclerosis, external bleeding, and urticaria [7,8]. Though Cortex Dictamni is widely used, some studies have reported that some Chinese patented medicines or compounds derived from Cortex Dictamni induce liver toxicity in recent years, such as Compound Qingdai pills [9], Xiaoyin tablets [10], Keyin pills [11], Vitiligo capsules [12], and Zhixue capsules [13]. In the 17th issue of the “ADR Report” in 2008, the National Centre for Monitoring Adverse Drug Reactions received many reports of liver damage induced from Zhixue capsules [14]. Furthermore, there were some studies related to the toxicity of Cortex Dictamni. Liu reported that 20 patients treated with Cortex Dictamni induced liver injury [15], Huang reported that a typical clinical case of taking Cortex Dictamni powder caused liver damage [16]. 

We found no reports of toxicity on Cortex Dictamni in ancient TCM literature, but pharmacological studies have shown that Cortex Dictamni contains alkaloids, saponins, and other chemical compounds which may increase its toxicity risk [17]. However, to date, very few investigations have been conducted regarding the toxicity of Cortex Dictamni, indicating the lack of direct and reliable research evidence on this issue. In our previous study, we reviewed the published papers about Zhixue capsules and conducted research on its liver toxicity. We conducted research on possible drug-related factors and concluded that Cortex Dictamni could be the main contributor to liver damage. Meanwhile, we also found that the most important factors related to hepatotoxicity are technological factors [18,19]. According to the literature, both CDAE and CDEE were often applied in clinic, such as in the treatment of skin inflammation, tinea, rheumatoid arthritis, coronary atherosclerosis, external bleeding, and neuroprotection [7,20]. Furthermore, there are no studies regarding the toxicity of CDEE. Thus, here we conducted the study in vivo and in vitro hepatotoxicity of CDAE and CDEE, which could provide valuable data for clinical drug safety research of Cortex Dictamni. In present-day researches, animal models and in vitro models are mainly used in the safety evaluation of herb-derived compounds. One study suggested that it is not very accurate to predict DILI in animal models because the hepatotoxicity induced by drugs is a complex process which could result in multiple endpoints. With the development of many novel in vitro approaches, we are now able to address the multiple mechanisms involved in hepatotoxicity [21]. In the past few years, high content analysis (HCA) technology based on cellular analysis has emerged as a powerful technology for evaluating multiparametric assays on their ability to predict factors related to hepatotoxicity, which may help to understand the mechanisms of hepatotoxicity. In this study, we first evaluated the hepatotoxicity of CDAE and CDEE in mice, and then compared the chemical constituents between CDAE and CDEE to choose the major compounds for further studies. We further used CCK-8 assays to evaluate the cell viability following 48 h of compounds exposure and determined the potential toxic compounds. In order to explore the possible mechanism of its hepatotoxicity, we used the following HCA parameters for our study. Cell number, nuclear intensity, mitochondria membrane potential (MMP), cell membrane permeability, and concentration of reactive oxygen species (ROS). Ultimately, these research results will provide new data on the clinical safety of Cortex Dictamni and help guide rational drug use in clinical applications and successfully develop new preparations.

## 2. Results

### 2.1. Subchronic Toxicity in Mice

#### 2.1.1. General Clinical Signs

No significant differences in body weight and food consumption were observed between the drug-treated groups and the control group, as shown in Table 1.

No drug-related deaths and toxicity signs (such as hair loss, hypoactivity, sluggish reaction, or diarrhoea) were observed in the drug-treated groups during the administration period.

#### 2.1.2. Serum Biochemistry

Table 2 shows the values of the biochemical parameters at the end of the administration periods. Compared with the control group, the male mice group giver 9.2 g/kg of CDEE with ALT and AST concentrations significantly increased (*p* < 0.05). Meanwhile, significant increases in ALB and TP were observed in male mice treated with CDEE at a dose of 9.2 g/kg (*p* < 0.05, *p* < 0.01). In the groups with 9.2 g/kg dose CDAE and CDEE, TG significantly increased (*p* < 0.05, *p* < 0.01,). In female mice with the 9.2 g/kg CDEE, ALT and AST significantly increased (*p* < 0.01, *p* < 0.05). ALB, GLB and TP increased in the CDAE and CDEE-treated group with 4.6 g/kg and 9.2 g/kg dose compared to the control group (*p* < 0.01, *p* < 0.05).

#### 2.1.3. Morphological Analysis

Table 3 shows the values of liver-related indexes at the end of the administration periods. In males treated with a high dose of CDEE, the absolute liver weight, relative liver weight, and liver/brain weight ratio were significantly increased compared with the control group (*p* < 0.05, *p* < 0.01). Meanwhile, the absolute liver weight, relative liver weight, and liver/brain weight ratio in the high-dose CDAE and CDEE groups were significantly elevated in female mice (*p* < 0.05, *p* < 0.01) compared to the control group.

**Figure 1:**
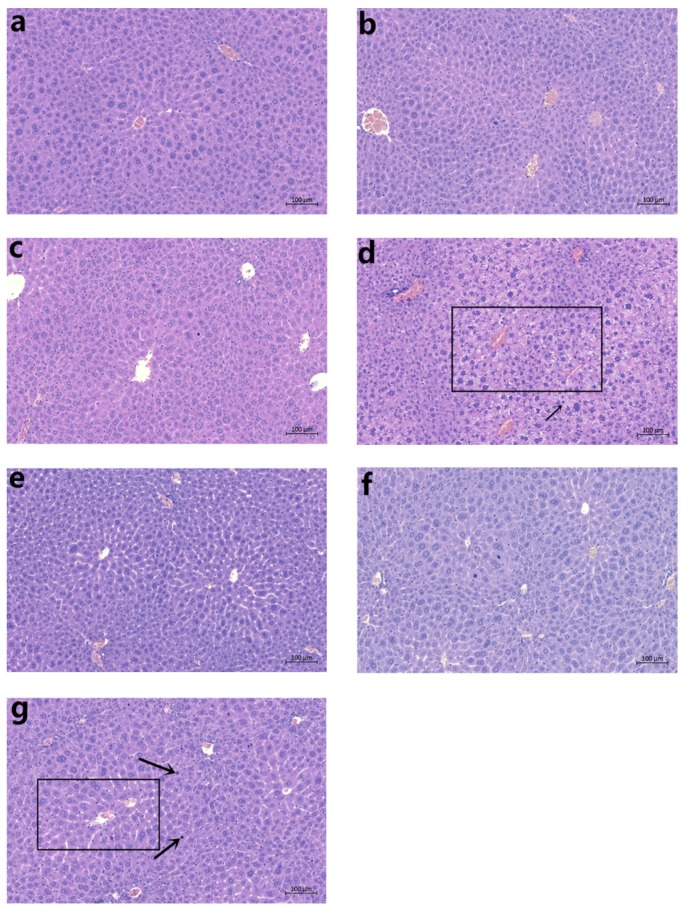
Histopathological examination of the livers of mice treated with the control (**a**); CDAE 2.3 g/kg (**b**); CDAE 4.6 g/kg (**c**); CDAE 9.2 g/kg (**d**); CDEE 2.3 g/kg (**e**); CDEE 4.6 g/kg (**f**); and CDEE 9.2 g/kg (**g**) for 28 days. (HE, 300×).

Observations of hepatic tissue sections showed that the high-dose CDAE and CDEE groups had nuclear shrinkage, suggesting apoptosis. Cellular enlargement was also observed in these groups (Figure 1).

### 2.2. Comparison of Composition Difference between CDAE and CDEE 

The results of the subchronic toxicity test in mice showed that both CDAE and CDEE had potential hepatotoxicity and that CDEE may be more toxic than CDAE. It is speculated that the active components of Cortex Dictamni inducing the toxicity would be enriched in alcohol extract. Thus, the ingredients differences between CDEE and CDAE were investigated in order to find out the active components of Cortex Dictamni for potential toxicity using HPLC.

**Figure 2:**
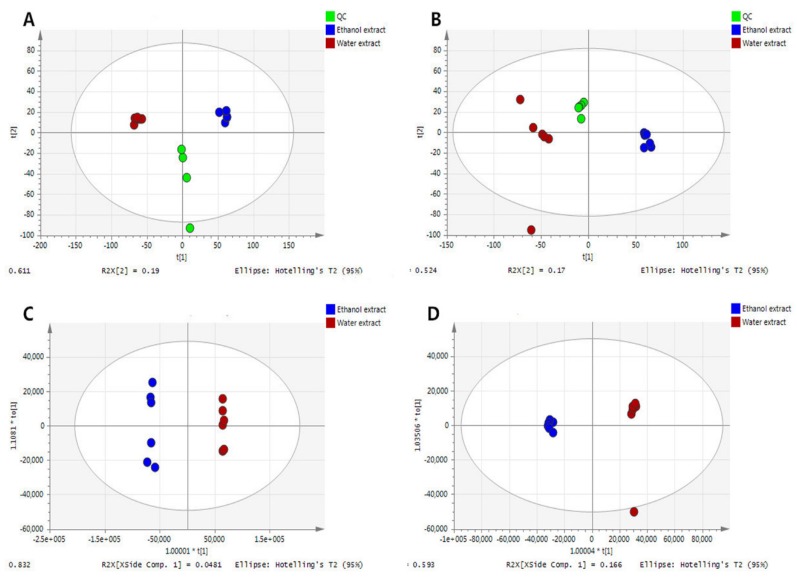
Partial Least Squares Discriminant Analysis (PLS-DA) diagram of CDAE and CDEE. (**A**) Positive ions (PCA); (**B**) Negative ions (PCA); (**C**) Positive ions (PLS-DA); (**D**) Negative ions (PLS-DA).

Chemical constituents in CDAE and CDEE were systematically analysed using the HPLC-Orbitrap MS. After the original mass spectrometry data were processed by SEIVE 2.1, a total of 5000 common components were obtained from the original mass spectrometry data of CDAE and CDEE. After normalisation and standardisation, the data were imported into SIMCA-P 13.0 software for multidimensional statistical analysis. As shown in Figure 2A,B, the results showed that all the samples were grouped into two categories using the principal component analysis method (PCA). CDAE and CDEE had good separation degree (R2X > 0.80, Q2 > 0.85, Eigen value < 1.5), indicating that different extraction could cause significant changes in relevant components. In order to explore the internal components inducing the difference between these two extracts, the Orthogonal Projections to Latent Structures Discriminant Analysis (OPLS-DA) model discrimination method was used to analyse each group (Figure 2C,D). The card value screening was conducted according to the variable differentiation in the projection (VIP). More than 30 different components were obtained between CDAE and CDEE (VIP > 1 and *p* < 0.05). Among them, dictamnine, obakunone, fraxinellone, and limonin, which have the most remarkable difference, were further studied.

**Figure 3:**
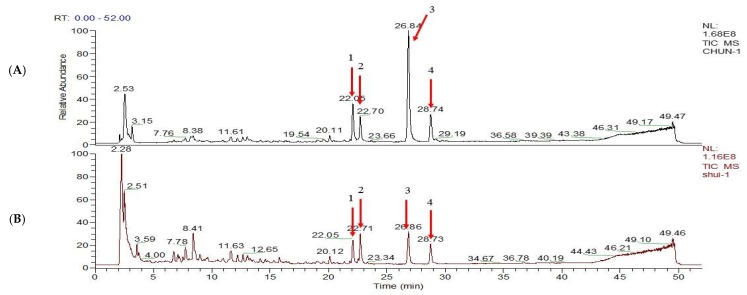
Positive ion model diagram of CDEE (**A**) and CDAE (**B**). 1. Dictamnine 2. Limonin 3. Obakunone 4. Fraxinellone.

The absolute content of dictamnine, obakunone, fraxinellone, and limonin in CDAE and CDEE was further determined in Figure 3. The results showed that the contents of dictamnine, obakunone, fraxinellone in CDEE were 2.27, 5.28, and 2.15 times of CDAE as shown in Table 4.

### 2.3. In Vitro Validation of Toxic Components of Cortex Dictamni

#### 2.3.1. CCK-8 Assay

**Figure 4:**
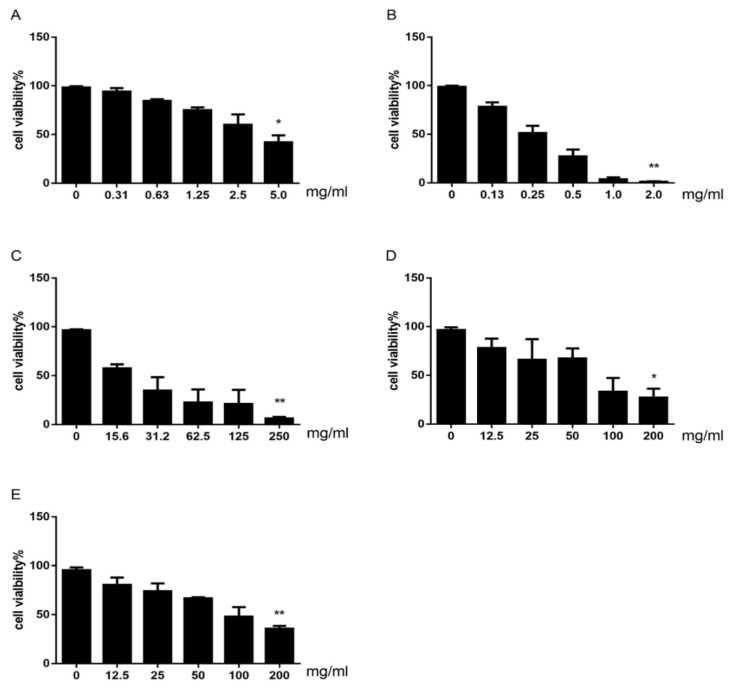
Cell viability of HepG2 cells treated with CDAE (**A**); CDEE (**B**); dictamnine (**C**); obakunone (**D**); and fraxinellone (**E**) for 48 h, as estimated by the CCK-8 assay (Values are expressed as means ± SDs, * *p* < 0.05, ** *p* < 0.01 compared with the control group. *n* = 3).

In order to determine the toxic constituents in Cortex Dictamni, we used the CCK-8 assay to estimate the potential cytotoxic effect of compounds. The results in Figure 4 demonstrated the inhibitory effects of different doses of CDAE and CDEE on HepG2. CDAE and CDEE decreased the viability of HepG2 cells in a dose-dependent manner after treatment for 48 h. Exposure of HepG2 cells to CDAE and CDEE at the highest dose resulted in significantly fewer viable cells compared to control cells (*p* < 0.5, *p* < 0.01). Meanwhile, the data showed that CDEE was more toxic than CDAE. Furthermore, exposure of HepG2 cells to dictamnine, obakunone, and fraxinellone at the highest dose significantly decreased the viability (*p* < 0.01, *p* < 0.5, *p* < 0.01). It was found that dictamnine had stronger toxicity than other two constituents. The three compounds were used for further studies of hepatotoxicity mechanisms.

#### 2.3.2. HCA Assay

**Figure 5:**
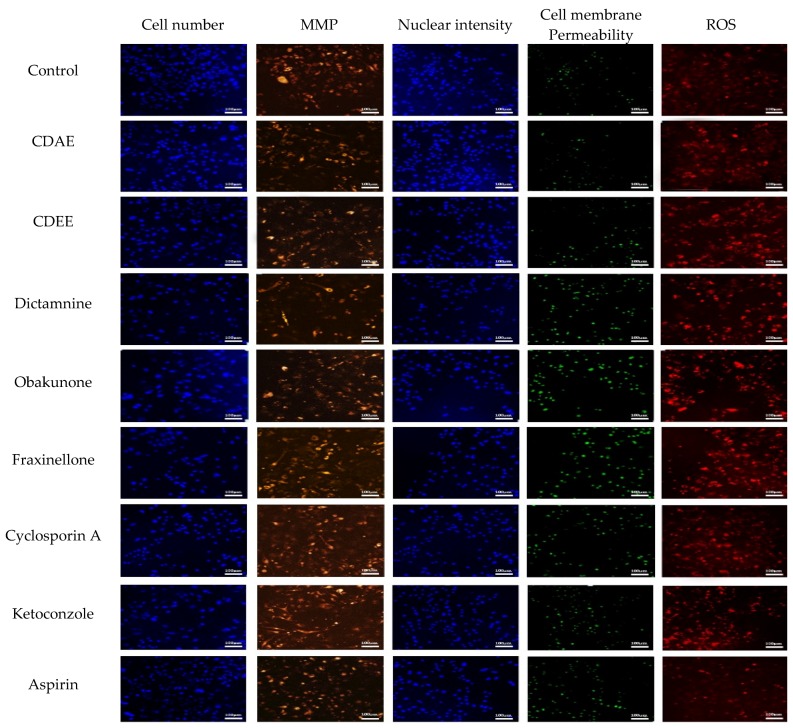
Representative images of HepG2 cells incubated with CDAE and CDEE using a high content imaging assay. The scale bar corresponds to 100 μm.

**Figure 6:**
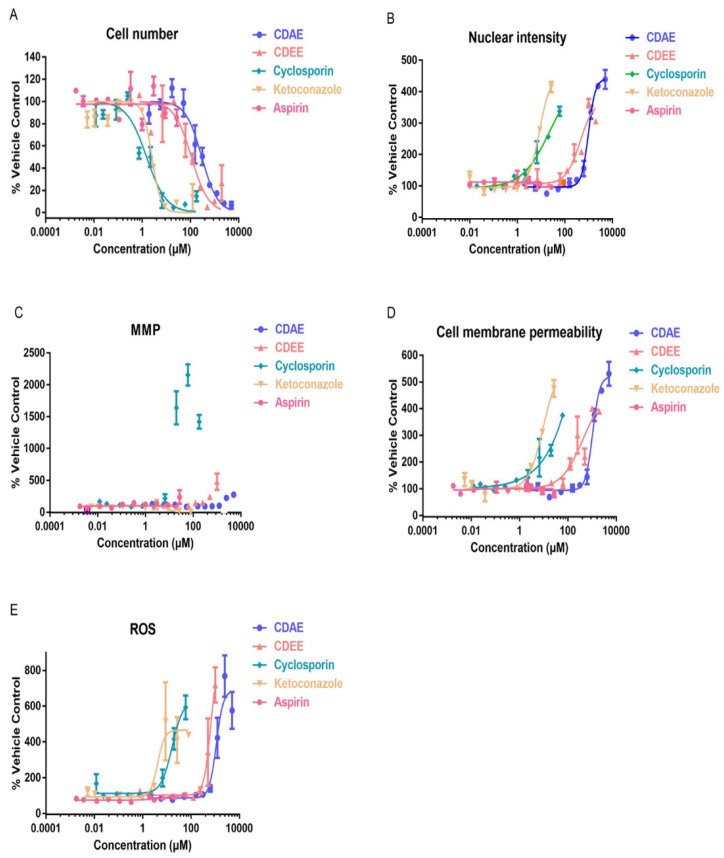
Estimation of drug-induced liver toxicity of CDAE and CDEE using a high content imaging assay. (**A**) Cell number; (**B**) nuclear intensity; (**C**) mitochondria membrane potential (MMP); (**D**) cell membrane permeability; and (**E**) reactive oxygen species (ROS).

**Figure 7:**
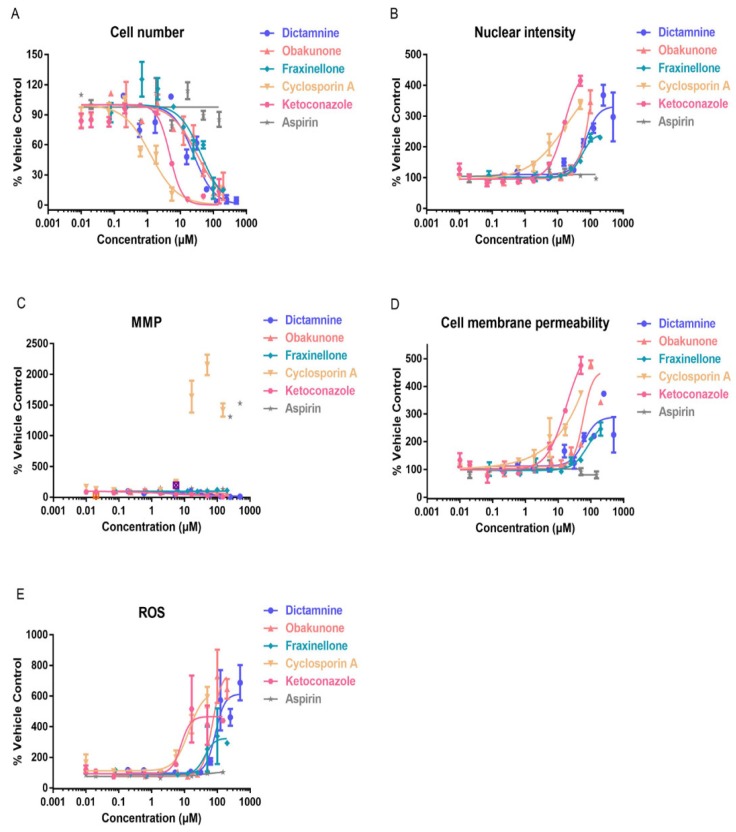
Estimation of drug-induced liver toxicity of dictamnine, obakunone, and fraxinellone using a high content imaging assay. (**A**) Cell number; (**B**) nuclear intensity; (**C**) mitochondria membrane potential (MMP); (**D**) cell membrane permeability; and (**E**) reactive oxygen species (ROS).

To further explore the mechanisms of hepatotoxicity of these components, the multiparameter cytotoxicity assay endpoints were evaluated in HepG2 cells following 48 h of compounds exposure using HCA (Figure 5). As shown in Figure 6 and Figure 7, the parameters used to evaluate the toxicity were the cell number, nuclear intensity, MMP, cell membrane permeability, and ROS. The IC_50_ values of extracts and compounds of these parameters were shown in Table 5. Meanwhile, the data showed us that all of the five parameters might be potential mechanisms that induce toxicity into HepG2 cells (Table 6).

## 3. Discussion

DILI is a major concern during drug development, and the evaluation of potential hepatotoxicity of drugs plays a key role in drug development and use. The toxicity of TCM herbs is an important concern of TCM practice as the premise and guarantee TCM safety.

Cortex Dictamni was recorded for the first time in “Shen Nong’s Herbal Classic”, which told little about its toxicity. However, in recent years, the toxicity of some formulations containing Cortex Dictamni have been frequently reported, such as Qingdai pills, Keyin pills, Vitiligo capsules, and Zhixue capsules. Furthermore, there have been studies related to the toxicity of Cortex Dictamni. Jang reported four cases of Cortex Dictamni decoction-induced toxic jaundice hepatitis [22]. McRae reported that Cortex Dictamni caused six cases of hepatitis [23]. However, until now, limited safety evaluation data have been available with respect to the clinical use of Cortex Dictamni. 

In the Chinese pharmacopoeia, the recommended highest daily dose of Cortex Dictamni was 10 g (0.17 g/kg for a 60 kg human). However, the clinical application of Cortex Dictamni complex prescription has been raised; the commonly used daily dosage is 15 g or 30 g, sometimes it could even reach the dosage of 60 g. In this study, Cortex Dictamni, in quantities of 2.3, 4.6, and 9.2 g/kg/day were given to mice to estimate the human equivalent doses of 15, 30, 60 g/day, respectively. Ultimately, these research results could provide new data on the clinical safety of Cortex Dictamni and help guide rational drug use in clinical applications. Furthermore, we found that previous studies have mainly involved the toxicity of the aqueous extract of Cortex Dictamni, but the ethanol extract is often used in clinic, such as Zhixue capsules. In our previous studies, we have found that the main reason for the hepatotoxicity of Zhixue capsules was related to the ethanol extraction process, and Cortex Dictamni was the main drug factor. Thus, in this study, we compared the two different extracts of Cortex Dictamni. 

We used two different dimensions of extracts and doses of Cortex Dictamni to evaluate the safety of the drug, combining the gold standard of histopathology with the indexes of organ and blood biochemistry to evaluate the toxicity of Cortex Dictamni comprehensively. This is of important clinical significance as it provides a base for drug safety. 

Firstly, we evaluated the toxicity of Cortex Dictamni using animal models. Changes in general behaviours and body weight have previously been used as important indicators in toxicity evaluations [24,25]. In the current study, no significant changes in general behaviour and body weight were observed between drug-treated groups and the control group, suggesting that Cortex Dictamni does not affect the normal growth of mice. We are told that the liver and kidneys are frequent targets of drug action. The liver is used for drug biotransformation, while the kidneys are used for drug excretion [26]. The changes in serum ALT and AST are the most common biomarkers used to predict liver damage [27,28]. In our study, their levels were significantly increased among the treatment groups, suggesting that subchronic treatment with CDAE and CDEE does cause severe liver injury, which prompts further research on this issue.

The relative organ weight is regarded as an indicator of toxic effect [29]. An increase in the organ:body weight ratio is an indication of inflammation [30]. In the present study, the absolute liver weight, relative liver weight, and liver/brain weight ratio increased significantly in the mice treated with a 6.4 g/kg dose of CDAE or CDEE, suggesting induced liver damage. Furthermore, observations of pathological sections showed that in the high-dose CDAE and CDEE groups, the mice showed nuclear shrinkage. Meanwhile, cellular enlargement was also observed in the high-dose CDAE and CDEE groups. 

The observations above demonstrated that there was hepatotoxicity in mice treated with 9.2 g/kg of CDAE or CDEE, which is equivalent to 60 g/kg in humans; this dosage is also used in some diseases. Besides, in the present study, the period of drug administration is equivalent to one week of clinical medication, but the actual course of drug usage of Cortex Dictamni is longer than one week. As far as we know, the toxicity of drugs is closely related to the dosage and duration of medication. Thus, the results suggested us that ethanol extraction process could increase the toxicity of Cortex Dictamni, and more close observation to the clinical use of Cortex Dictamni shall be paid in the future.

Secondly, in order to find out the possible toxic material basis of Cortex Dictamni, we identified the different constituents of CDAE and CDEE using HPLC-MS/MS. The results revealed that the main components of CDAE and CDEE were dictamnine, obakunone, fraxinellone, and limonin, and the contents of dictamnine, obakunone, fraxinellone, and limonin in CDEE were 2.27, 5.28, 2.15, and 1.33 times of CDAE, respectively. Thus, the possible toxic ingredients maybe enriched in CDEE, which could be the reason for having higher toxicity. Besides, limonin is often used as food additive. Therefore, dictamnine, obakunone, and fraxinellone were determined as the candidates of toxic compounds contained in the extracts of Cortex Dictamni. In recent years, some studies have reported that animal models cannot adequately predict human liver toxicity, possibly due to differences in the immune system, the metabolic capacities, and genetic sensitivities between animals and humans [31]. Thus, a cell-based assay could provide a better predictive power for toxicity research. HepG2 cells display cellular functions similar to normal hepatocytes; therefore, the HepG2 cell line is typically chosen as a suitable model for drug safety assessments [32]. In the in vitro studies, CCK 8 and HCA experiments were used to evaluate the potential hepatotoxicity of Cortex Dictamni in HepG2 cells.

The CCK-8 assay is a useful technology for estimating the potential cytotoxicity in vitro for further research of DILI screening [33]. The results here suggested that CDAE and CDEE decrease the cell viability of HepG2 cells in a dose-dependent manner with CDEE more toxic than CDAE. Meanwhile, dictamnine, obakunone, and fraxinellone decreased cell viability in a dose-dependent manner after treatment for 48 h in this assay. However, they also demonstrated that certain conventional cytotoxicity assays are sometimes in poor concordance with human toxicity.

In order to obtain more information about the mechanisms of hepatotoxicity, HCA was chosen, since it may have more endpoints and is considerably more reliable [34,35]. Technically speaking, HCA is a high-throughput, imaging-based cellular analysis technology which can simultaneously detect changes in cell growth, morphology, apoptosis, and signal transduction at the same time for toxicology research. At present, the main known mechanisms of hepatotoxicity induced by Chinese medicine are direct cytotoxicity, cell membrane damage, and mitochondrial damage [36,37].

In our present study, we studied five parameters: the cell number, nuclear intensity, mitochondria membrane potential, cell membrane permeability, and concentration of reactive oxygen species. These five indicators reflect the basic mechanisms of cell apoptosis and death in different angles of hepatotoxicity. The cell number can be used to evaluate the degree of cell injury, which is related to cell death. Nuclear intensity mainly evaluates the occurrence of nuclear shrinkage and cell apoptosis. Cell membrane permeability is mainly used to reflect the integrity of cells, whose increase indicates the disruption of ionic gradients or mitochondrial dysfunction [38]. Oxidative stress results from an imbalance between reactive oxygen species (ROS) formation in cells and antioxidant activity, as it plays a role in the hepatotoxicity mechanism. An increased level of ROS can damage lipids, proteins, or DNA, leading to lipid peroxidation and mitochondrial dysfunction [39]. MMP mainly detects the potential differences between the inner and outer membranes of mitochondria, which is a frequently used indicator for the early evaluation of potentially hepatotoxic drugs [40,41]. Mitochondria play important roles in cell survival and the stable maintenance of intracellular environments. Changes in mitochondria are regarded as important morphological changes in apoptosis and have been recognised as early cell markers of apoptosis. In our present study, the cell number decreased, while the nuclear intensity, mitochondria membrane potential, cell membrane permeability, and concentration of reactive oxygen species increased after CDAE and CDEE exposure, suggesting that hepatotoxicity induced by CDAE and CDEE may lead to cell apoptosis.

Furthermore, the cell number decreased, while the nuclear intensity, cell membrane permeability, and concentration of ROS were shown to increase, meanwhile, mitochondrial membrane potential also changed in HepG2 cells following 48 h of compounds exposure using HCA, suggesting that hepatotoxicity induced by Cortex Dictamni may lead to cell apoptosis. The results of the cell viability assay and the IC_50_ values obtained from the HCA assay indicated that dictamnine, obakunone, and fraxinellone were all cytotoxic to hepatocytes. We found that dictamnine may have stronger toxicity when comparing the IC_50_ values in HCA.

Nowadays, experts on clinical issues have proposed causes of the toxicity of Chinese herbal medicine, such as the lack of theoretical guidance of Chinese medicine, the ignorance of the compatibility of traditional Chinese medicine, and improper use of dosages. The pharmacological and toxicological effects of toxic ingredients have also become fundamental measures to control the toxicity risks of Chinese herbal medicines. Cortex Dictamni mainly contains alkaloids, lactones, and sesquiterpene glycosides. Recent studies have reported that limonin, dictamnine, obacunone, and fraxinellone are the four main constituents of Cortex Dictamni extract [42,43] that have the potential to increase toxicity risk. It was said that the dictamnine contained in Cortex Dictamni has certain toxic effects on HepG2 hepatocytes and the livers of animals [44]. Our present research were consistent with these previous studies, moreover, we illuminated the possible mechanisms of hepatotoxicity of Cortex Dictamni. Though high-content screening technology shows a highly efficient and convenient system for predicting human DILI, it still has limitations. The cytotoxicity evaluation is based on the single-cell level, while the whole-organism metabolism and immune system is much more complex. Therefore, in vitro studies do not accurately represent the liver in vivo. With the development of molecular technology, there are some liver culture platforms, such as 3D liver culture model and microfluidic liver chips which have been developed to recapitulate in vivo physiological conditions to enhance hepatocyte functions for assessing the toxicity of drugs [45,46]. Thus, further studies are needed to evaluate the toxicity of Cortex Dictamni as whole.

## 4. Conclusions

In conclusion, the results demonstrated that CDAE and CDEE have potential hepatotoxicity, and alcohol extraction process could increase toxicity. Dictamnine, obakunone, and fraxinellone may be the possible toxic components in Cortex Dictamni with dictamnine as the most potentially hepatotoxic component. Moreover, the potential mechanism of hepatotoxicity may be associated with changes in the cell number, nuclear intensity, mitochondria membrane potential, cell membrane permeability, and concentration of reactive oxygen species, which may induce cell apoptosis. Systematic research on common adverse drug reactions in clinical practice has always been an important issue in the field of traditional Chinese medicine. Our study provides applicable strategies for the safety research of many other herbal medicines. Furthermore, in future research, we seek to find more evidence for the appearance of clinical adverse reactions of Cortex Dictamni and to study the intrinsic mechanisms of toxicity, which may safeguard clinical usage of traditional Chinese herbal medicine based on the scientific research data.

## 5. Materials and Methods

### 5.1. Chemicals and Reagents

Organic solvents and reagents were all of HPLC grade. Acetonitrile was purchased from Merck (Darmstadt, Germany) and dimethyl sulfoxide (DMSO) was purchased from Sigma-Aldrich (St. Louis, MO, USA). Deionised water was prepared in a Milli-Q laboratory water purification system (Millipore, Bedford, MA, USA). Dictamnine, obakunone, and fraxinellone were purchased from Yuanye Bio-Technology Co. Ltd. (Shanghai, China). HepG2 cells were purchased from China Infrastructure of Cell Line Resources (Beijing, China). The ToxInsight DILI cartridge was acquired from Thermo Fisher Scientific (Waltham, MA, USA). Dulbecco’s modified Eagle’s medium (DMEM) and foetal bovine serum (FBS) were purchased from Gibco (Grand Island, NY, USA). Penicillin, streptomycin, cyclosporine A, and aspirin were obtained from Sigma Aldrich (Saint Louis, MO, USA). The cell counting kit (CCK-8) was from Dojindo (Tokyo, Japan). Liquid paraffin and neutral formalin were purchased from Beijing Chemical Works (Beijing, China).

### 5.2. Plant Material and Extraction

Cortex Dictamni (Bai-Xian-Pi) from the root bark of *Dictamnus dasycarpus* Turcz. (Rutaceae), was purchased from Beijing Yan Bei decoction Chinese Herbal Pieces Co., Ltd. (Beijing, China) and identified by Prof Xiangri Li (School of Chinese Materia Medica, Beijing University of Chinese Medicine).

For preparation of the aqueous and ethanol extracts, 10 kg of Cortex Dictamni decoction pieces were boiled with 80 L of distilled water or 85% ethanol under reflux for 2 h, twice. The decoction was filtered and then the filtrates were combined and concentrated using a rotary evaporator and were lyophilised under reduced pressure. The yields of these extract were approximately 31.7% (*w*/*w*) and 8.2% (*w*/*w*). The dried residue was stored at 4 °C and was resuspended in distilled water for use. 

### 5.3. Sample Preparation for UPLC Analysis 

The aqueous and ethanol extracts of the Cortex Dictamni (15 g) were boiled in 120 mL of deionised water or 85% ethanol under reflux for 2 h, twice. The filtrates were combined and concentrated under vacuum and then filtered with a 0.45-μm membrane filter before use. 

A few milligrams of dictamnine, obakunone, fraxinellone, and limonin were dissolved in 10 mL of methanol. 1 mL of these compound solutions was mixed into a 10 mL volumetric flask, and the mixturefiltered with a 0.45-μm membrane filter before use. 

### 5.4. UPLC-MS/MS Analysis

UPLC analysis was performed on a Thermo Scientific Dionex UltiMate 3000 UHPLC system (Santa Clara, CA, USA) equipped with an autosampler, a vacuum degasser unit, a quaternary pump, a diode array detector (DAD), and a column compartment. Samples were separated on a ZORBAX SB-C18 column (250 mm × 4.6 mm, 5 μm) at 35 °C. The mobile phase consisted of 0.1% formic acid (A) and acetonitrile (B), and the elution gradient was set as follows: 5–53% B (0–20 min), 53–68% B (20–40 min), 68–80% B (40–42 min), 80–90% B (42–47 min), 90–95% B (47–47.1 min), and 95% B (47.1–52 min). The flow rate was set at 1.0 mL/min, and the injection volume was 10 µL. ESI-MS analyses were carried out on a Thermo Fisher Scientific LTQ-Orbitrap XL hybrid mass spectrometer (Santa Clara, CA, USA) equipped with an electrospray ionization interface and an HESI-II source. Samples were analysed in negative ion mode, and full-scan mass spectra were acquired in a mass range of *m/z* 50 to 1000 Da. The resolving power was 30,000 for the full scans and 15,000 for the MS2 scans. The spray and capillary voltages were set to 4.0 V and 25.0 V, respectively; the tube lens was set to 110 V; and the source temperature was set to 300 °C. Nitrogen (purity > 99.99%) was used as both the sheath gas (30 units) and auxiliary gas (10 units).

High-resolution MS data processing was carried out using the QualBrowser of Xcalibur 2.1 (Thermo Fisher Scientific), including EIC, calculation of elemental compositions using potential metabolite ions with 5-ppm mass tolerance and isotope pattern simulation. By comparison to data regarding reference substances and by referring to the relevant literature, the components of each extract were identified, and the structure and composition of these components were determined.

### 5.5. Experimental Animals

Adult male and female ICR mice (18–22 g) were obtained from the Beijing SPF Animal Technology Company (Beijing, China). The mice were housed in groups of matched weights in certain humidity (40–70%) and temperature (20–25 °C) controlled room and were housed in a 12-h light/dark cycle environment during the tests. Food and water were available ad libitum. The study was approved by the experimental animal ethics subcommittee of Beijing University of Chinese Medicine (BUCM), the ethical review number is BUCM-4-2017122501-4037.The protocol was in accordance with the National Institute of Health Guide for the Care and Use of Laboratory Animals. In total, 140 mice were used in the study. 

### 5.6. Subchronic Toxicity Study

#### 5.6.1. Experimental Design

The tests were performed according to the Guideline on Repeated Dose Toxicity (CFDA, 2014). The ICR mice were weighed and randomly divided into seven groups (one control group and six treated groups), with 10 males and 10 females in each group. The control group was orally administered distilled water and the treated groups were administrated CDAE or CDEE, respectively, at doses of 2.3, 4.6, or 9.2 g/kg/day, which were set as the low-, medium-, and high-dose groups, respectively. The mice were administered with the same volume of 10 mL/kg/day based on their body weight during the 28-day treatment period. 

Clinical observations, food consumption, body weights, biochemical parameters, organ weights, and the histopathology of mice were examined at the end of the 28-day treatment period.

#### 5.6.2. General Clinical Signs, Body Weights, and Food Consumption

The general clinical signs of the mice, such as general activity, behaviour, appearance, hair lustre, and urine, were observed after daily administration. Body weight and food consumption were measured weekly.

#### 5.6.3. Biochemical Analysis

The blood samples were collected from the mice ocular vein. Serum was obtained by centrifugation of whole blood at 3500 r/min for 10 min. The parameters of the biochemistry assays: alkaline phosphatase (ALP), alanine transaminase (ALT), aspartate transaminase (AST), direct bilirubin (DBIL), total bilirubin (TBIL), albumin (ALB), globulin (GLB), total protein (TP), high density lipoprotein cholesterol (HDLC), low density lipoprotein cholesterol (LDLC), triglyceride (TG), and cholesterol (CHO).

#### 5.6.4. Morphological Study

At the end of the administration, necropsies were performed on all animals. The organ weights of the liver and brain were measured. The ratio of each organ weight to the terminal body weight in grams per 100 g body weight (relative organ weight) was calculated, respectively. 

The livers were fixed in 10% neutral buffer formalin and embedded with paraffin. Subsequently, samples were sliced into 3 µm thickness using a rotary microtome. Then the sections were stained with haematoxylin–eosin (HE) After staining, the slides were observed and examined using an optical microscope at 300× magnification (Carl Zeiss Meditec AG, DE). 

### 5.7. Cell Culture

Cells were cultured in DMEM medium supplemented with 10% (*v*/*v*) of FBS and 1% antibiotics (penicillin, streptomycin) at 37 °C in a humidified incubator containing atmospheric air and 5% CO_2_. The cells were subcultured when they were 80–90% confluent.

### 5.8. CCK-8 Colorimetric Assay

Cells in the logarithmic growth phase were selected for the experiment. The viability of HepG2 cells after treatment with CDAE and CDEE was measured using CCK-8 assay. Exponentially-growing HepG2 cells were seeded into 96-well plates at a density of 5 × 10^4^ cells/mL in a final volume of 100 µL medium for 24 h. Then, the cells were treated with varying concentrations of CDAE or CDEE. After exposure for 48 h, 10 µL of CCK-8 solution was added to each well for 3 h. The optical density at 450 nm was measured for each well using an absorbance microplate reader (Thermo, Waltham, MA, USA). Percentage (%) = (OD of experimental sample/OD of control sample) × 100%. The concentrations of CDAE were 5, 2.5, 1.25, 0.63, and 0.31 mg/mL, which were diluted in the culture medium, while the CDEE stock solution was prepared in DMSO and was serially diluted in the culture medium for different concentrations (2, 1, 0.5, 0.25, and 0.13 mg/mL). Dictamnine, obakunone, and fraxinellone were also prepared in DMSO. Dictamnine was serially diluted in the culture medium for different concentrations (250, 125, 62.5, 31.2, 15.6 μM), while obakunone and fraxinellone were serially diluted in the culture medium for different concentrations (200, 100, 50, 25, 12.5 μM). The final DMSO concentration in the culture medium never exceeded 0.5% (*v/v*).

### 5.9. HCA Assay

In this assay, we used five parameters to determine the drug-induced liver toxicity: cell number, nuclear intensity, MMP, cell membrane permeability, and concentration of ROS. The exponentially-growing HepG2 cells were seeded into 384-well plates at a density of 1 × 10^5^ cells/mL in a final volume of 20 µL medium for 24 h. Then, the cells were treated with varying doses of CDAE or CDEE. After 48 h incubation, the cells were applied to a fluorescent dye cocktail, which contained SYTOX Green Nucleic Acid Stain for Cell Membrane Permeability, Cell ROX Deep Red Reagent for ROS, TMRM for MMP, and Hoechst 33342 to determine the cell number or nuclear intensity. The excitation and emission wavelengths were 346 nm and 460 nm, respectively. The signal of the cell membrane permeability dye was acquired with 504 nm excitation and 523 nm emission wavelengths. For the ROS signal collection, the excitation and emission wavelengths used were 644 nm and 655 nm, respectively. The mitochondrial signal (MMP) was detected at 540 nm excitation and 580 nm emission wavelengths. The images were observed and examined using 10 × objective. The concentrations of CDAE and CDEE were the same as that of CCK-8 assay. The positive control compounds were cyclosporin A and ketoconazole, and the negative control compound was aspirin. DMSO (0.5%) was used as Vehicle control.

- For the cell number, % vehicle control = (cell number compound/cell number vehicle) × 100%
- For the nuclear intensity, mitochondria membrane potential, reactive oxygen species, and cell membrane permeability, % vehicle control = (read compound/read vehicle) × 100%
- To determine the IC_50_ of the cell number and mitochondria membrane potential, the % vehicle control was plotted against the concentration of test compounds and the data was fitted to a sigmoidal dose–response curve with a variable slope using GraphPad Prism 5.0. The IC_50_ of the compound was calculated from the curve as Y = Bottom + (Top − Bottom)/(1 + 10^((LogIC_50_ − X) × HillSlope)) Max Inhibition = (100% − % vehicle) at the concentration that showed the highest inhibition effect.
- To determine the EC_50_ of the nuclear intensity, reactive oxygen species, and cell membrane permeability, the % vehicle control was plotted against the concentration of the test compounds, and the data was fitted to a sigmoidal dose–response curve with a variable slope using GraphPad Prism 5.0 (GraphPad Software Inc., San Diego, CA, USA). The EC_50_ of the compound was calculated from the curve as Y = Bottom + (Top − Bottom)/(1 + 10^((LogEC_50_ − X) × HillSlope)) Max Induction = % vehicle at the concentration that showed the highest induction effect.

### 5.10. Statistical Analysis

The statistical analysis was performed using GraphPad Prism software (GraphPad Prism 5.0, version 2.0; GraphPad Software Inc., San Diego, CA, USA). The variance in data for body weight, serum biochemistry, and organ weights (both absolute and relative weights) was checked for homogeneity by Bartlett’s procedure. The data were assessed by one-way analysis of variance (ANOVA), for the data with equal variances, followed by Dunnett’s tests. In contrast, the data that demonstrated unequal variance were analysed using the Kruskal–Wallis test followed by Dunn’s multiple comparisons tests. Probability values lower than 5% (*p* < 0.05) were considered significant.

## Figures and Tables

**Table 1:** Effects of oral administration of CDAE and CDEE on body weights in mice.

Days	Control	CDAE (g/kg)			CDEE (g/kg)		
		2.3	4.6	9.2	2.3	4.6	9.2
Males							
Day 0	22.47 ± 0.99	22.47 ± 0.86	22.73 ± 1.14	22.53 ± 1.01	22.53 ± 0.84	22.28 ± 0.97	22.03 ± 1.07
Day 7	32.11 ± 1.68	31.19 ± 2.53	32.75 ± 1.79	32.20 ± 2.06	31.79 ± 1.52	31.06 ± 2.30	31.33 ± 2.28
Day 14	35.73 ± 2.32	35.90 ± 3.00	37.03 ± 2.51	36.31 ± 2.76	35.79 ± 2.29	36.30 ± 2.19	34.53 ± 2.96
Day 21	37.10 ± 3.44	36.80 ± 3.41	38.10 ± 2.87	37.96 ± 2.38	36.88 ± 2.60	37.95 ± 2.52	34.65 ± 3.40
Day 28	39.13 ± 2.47	38.21 ± 3.46	40.24 ± 3.18	39.56 ± 2.17	38.23 ± 3.52	38.61 ± 3.76	36.84 ± 5.42
Females							
Day 0	21.51 ± 0.95	21.17 ± 0.88	20.97 ± 0.99	21.49 ± 1.18	21.44 ± 0.85	21.35 ± 0.72	21.19 ± 1.24
Day 7	27.11 ± 1.97	26.05 ± 1.34	26.90 ± 1.75	26.26 ± 2.84	26.41 ± 2.13	26.19 ± 1.70	26.73 ± 2.34
Day 14	28.63 ± 2.41	27.82 ± 1.75	28.07 ± 1.46	28.37 ± 3.32	27.99 ± 1.98	27.54 ± 1.94	28.44 ± 2.34
Day 21	29.07 ± 2.65	28.65 ± 1.90	29.60 ± 1.61	29.75 ± 4.21	28.83 ± 2.62	28.09 ± 2.04	29.11 ± 2.56
Day 28	30.13 ± 2.80	29.04 ± 2.42	30.11 ± 1.94	30.14 ± 3.92	29.57 ± 2.42	28.53 ± 2.07	30.14 ± 3.12

Values are expressed as means ± SDs; *n* = 10.

**Table 2:** Effects of oral administration of Cortex Dictamni aqueous extract (CDAE) and Cortex Dictamni ethanol extract (CDEE) on the biochemical parameters in mice.

Parameters	Control	CDAE (g/kg)			CDEE (g/kg)		
		2.3	4.6	9.2	2.3	4.6	9.2
Males							
ALP (U/L)	96.33 ±13.59	72.33 ± 11.23	96.13 ± 20.98	112.7 ± 18.43	93.30 ± 14.35	104.5 ± 17.04	113.1 ± 24.89
ALT (U/L)	45.05 ± 13.92	39.73 ± 17.94	63.12 ± 38.54	64.12 ± 40.35	42.60 ± 7.77	48.70 ± 15.64	159.7 ± 160.3 *
AST (U/L)	120.7 ± 14.21	114.1 ± 13.24	125.4 ± 29.43	171.5 ± 24.44	131.9 ± 23.72	150.5 ± 25.35	215.6 ± 150.2 *
DBIL (μM)	0.65 ± 0.16	0.72 ± 0.04	0.64 ± 0.17	0.55 ± 0.22	0.75 ± 0.12	0.75 ± 0.10	0.71 ± 0.20
TBIL (μM)	4.29 ± 0.57	4.44 ± 0.40	4.13 ± 0.49	3.13 ± 0.37	3.96 ± 0.35	4.64 ± 0.53	4.10 ± 0.70
ALB (g/L)	32.19 ± 2.08	34.81 ± 0.50	33.47 ± 2.26	33.70 ± 1.99	34.09 ± 3.61	35.79 ± 1.40	36.73 ± 1.62 *
GLB (g/L)	24.79 ± 2.28	26.39 ± 1.47	25.26 ± 1.50	25.05 ± 1.36	26.36 ± 1.85	26.45 ± 0.63	27.15 ± 1.69
TP (g/L)	56.99 ± 4.14	61.20 ± 1.60	58.73 ± 2.79	58.75 ± 2.52	60.45 ± 4.86	62.24 ± 1.60	63.88 ± 2.95 **
HDLC (mM)	3.04 ± 0.36	3.02 ± 0.34	2.87 ± 0.43	2.69 ± 0.36	2.89 ± 0.42	3.21 ± 0.29	3.02 ± 0.82
LDLC (mM)	0.55 ± 0.09	0.58 ± 0.11	0.48 ± 0.13	0.36 ± 0.10	0.51 ± 0.08	0.57 ± 0.11	0.51 ± 0.14
TG (mM)	1.80 ± 0.29	1.63 ± 0.31	2.52 ± 0.53	2.41 ± 0.40 *	2.00 ± 0.43	1.83 ± 0.70	2.64 ± 0.50 **
CHO (mM)	1.82 ± 0.35	1.84 ± 0.25	1.61 ± 0.22	1.69 ± 0.22	1.80 ± 0.34	1.78 ± 0.40	1.90 ± 0.42
Females							
ALP (U/L)	130.0 ± 31.15	107.6 ± 26.31	112.8 ± 35.56	131.1 ± 26.87	103.1 ± 20.18	130.1 ± 28.10	124.7 ± 19.60
ALT (U/L)	30.63 ± 3.70	32.17 ± 5.54	46.28 ± 31.42	54.48 ± 18.34	31.52 ± 10.51	38.13 ± 10.70	69.50 ± 32.08 **
AST (U/L)	114.2 ± 10.73	120.0 ± 18.52	132.3 ± 38.01	143.1 ± 13.00	121.7 ± 19.71	129.3 ± 27.37	161.1 ± 30.92 *
DBIL (μM)	0.43 ± 0.11	0.39 ± 0.09	0.43 ± 0.09	0.44 ± 0.15	0.48 ± 0.13	0.44 ± 0.08	0.54 ± 0.17
TBIL (μM)	2.66 ± 0.28	2.28 ± 0.29	2.63 ± 0.28	2.59 ± 0.42	3.05 ± 0.25	2.83 ± 0.44	2.88 ± 0.38
ALB (g/L)	33.83 ± 1.84	35.89 ± 1.96	36.30 ± 1.65 *	36.63 ± 1.89 **	37.50 ± 1.02 **	38.38 ± 1.30 **	37.69 ± 1.63 **
GLB (g/L)	22.48 ± 1.28	23.22 ± 1.22	24.64 ± 2.69 *	24.46 ± 1.70 *	24.27 ± 1.37	25.54 ± 1.02 *	24.81 ± 1.60 *
TP (g/L)	56.31 ± 2.39	59.11 ± 2.85	60.93 ± 3.95 **	61.10 ± 3.42 **	61.77 ± 2.30 **	63.91 ± 2.11 **	62.49 ± 3.07 **
HDLC (mM)	2.03 ± 0.15	2.06 ± 0.26	1.98 ± 0.28	2.24 ± 0.35	2.16 ± 0.35	2.28 ± 0.25	2.46 ± 0.36
LDLC (mM)	0.35 ± 0.06	0.33 ± 0.08	0.29 ± 0.05	0.30 ± 0.08	0.31 ± 0.07	0.29 ± 0.05	0.31 ± 0.06
TG (mM)	1.94 ± 0.31	1.46 ± 0.14	1.54 ± 0.46	1.36 ± 0.55	1.41 ± 0.33	1.55 ± 0.20	1.59 ± 0.37
CHO (mM)	2.89 ± 0.25	2.88 ± 0.40	2.74 ± 0.40	3.01 ± 0.55	2.99 ± 0.53	3.08 ± 0.35	3.35 ± 0.52

Values are expressed as means ± SDs; *n* = 6; * *p* < 0.05, ** *p* < 0.01, compared with the control group.

**Table 3:** Effects of oral administration of CDAE and CDEE on liver-related indexes in mice.

Parameters	Control	CDAE (g/kg)			CDEE(g/kg)		
		2.3	4.6	9.2	2.3	4.6	9.2
Males							
absolute liver weight (g)	1.94 ± 0.24	1.75 ± 0.26	1.97 ± 0.32	1.87 ± 0.44	1.78 ± 0.23	1.90 ± 0.29	2.24 ± 0.28 *
relative liver weight (%)	4.94 ± 0.35	4.56 ± 0.38	4.89 ± 0.66	5.02 ± 0.72	4.63 ± 0.22	4.89 ± 0.39	5.67 ± 0.64 **
liver/brain (%)	4.11 ± 0.51	3.77 ± 0.42	4.25 ± 0.71	4.19 ± 1.01	3.81 ± 0.51	4.15 ± 0.59	4.96 ± 0.49 **
Females							
absolute liver weight (g)	1.34 ± 0.21	1.42 ± 0.21	1.44 ± 0.13	1.55 ± 0.22 *	1.34 ± 0.16	1.32 ± 0.15	1.53 ± 0.17 *
relative liver weight (%)	4.44 ± 0.48	4.91 ± 0.71	4.77 ± 0.33	5.13 ± 0.35 *	4.52 ± 0.33	4.61 ± 0.25	5.06 ± 0.27 *
liver/brain (%)	2.91 ± 0.40	3.08 ± 0.43	3.06 ± 0.28	3.40 ± 0.46 **	2.97 ± 0.38	2.93 ± 0.29	3.38 ± 0.26 **

Values are expressed as means ± SDs; *n* = 10; * *p* < 0.05; ** *p* < 0.01 compared with the control group.

**Table 4:** Comparison of contents of dictamnine, obakunone, fraxinellone, and limonin in CDAE and CDEE.

	Dictamnine				Obakunone		Fraxinellone			Limonin		
	Peak Area	Concentration (μg/mL)	Content (μg/g)	Peak Area	Concentration (μg/mL)	Content (μg/g)	Peak Area	Concentration (μg/mL)	Content (μg/g)	Peak Area	Concentration (μg/mL)	Content (μg/g)
Standard	231,191,845.5	11.00	——	323,906,511.5	60.00	——	56,264,623.5	60.00	——	41,016,446	10.00	——
CDAE 1	128,027,593	6.09	——	150,784,845	27.93	——	39,239,900	41.85	——	121,779,843	29.69	——
CDAE 2	136,755,354	6.51	——	145,896,477	27.03	——	34,745,736	37.05	——	128,391,047	31.30	——
Mean	132,391,473.5	6.30	104.99	148,340,661	27.48	457.97	36,992,818	39.45	657.48	125,085,445	30.50	508.27
CDEE 1	300,870,727	14.32	——	774,845,264	143.53	——	79,882,855	85.19	——	162,019,234	39.50	——
CDEE 2	299,295,542	14.24	——	791,742,097	146.66	——	78,950,339	84.19	——	170,064,825	41.46	——
Mean	300,083,134.5	14.28	237.96	783,293,680.5	145.10	2418.27	79,416,597	84.69	1411.48	166,042,029.5	40.48	674.70
CDEE/CDAE			2.27			5.28			2.15			1.33

**Table 5:** The IC_50_ values of test compounds and the control compound in HepG2 cells using HCA.

Compounds	Cell Number	Nuclear Intensity	Mitochondria Membrane Potential	Cell Membrane Permeability	Reactive Oxygen Species
CDAE	296.30	987.70	>5000	1064.00	1114.00
CDEE	109.70	439.20	>2000	449.20	668.90
dictamnine	25.46	72.12	34.43	61.47	87.70
obakunone	42.87	54.09	>200	84.00	41.51
fraxinellone	32.53	>200	>200	58.88	71.15
Cyclosporin A	1.27	20.07	>150	>150	13.75
Ketoconazole	4.83	15.96	6.15	17.95	7.87
Aspirin	>150	>150	>150	>150	>150

**Table 6:** Cytotoxicity of test compounds and the control compound in HepG2 cells using high-content analysis (HCA).

Compound	Cell Number	Nuclear Intensity	Mitochondria Membrane Potential	Cell Membrane Permeability	Reactive Oxygen Species	* of Positive
CDAE	+	+	+	+	+	5
CDEE	+	+	+	+	+	5
dictamnine	+	+	+	+	+	5
obakunone	+	+	−	+	+	4
fraxinellone	+	+	−	+	+	4
Cyclosporin A	+	+	+	+	+	5
Ketoconzole	+	+	+	+	+	5
Aspirin	−	−	−	−	−	0

The mean ± two SDs, mean ± three SDs, and mean ± five * SDs of the negative control (aspirin) data were used as the toxicity thresholds for the cell number, nuclear intensity, mitochondrial membrane potential, cell membrane permeability, and reactive oxygen species, respectively.
